# Clinical Outcomes and Safety of Ultra-Low-Dose Radiotherapy for Ocular Adnexal Lymphoma: A Systematic Review

**DOI:** 10.3390/cancers17172845

**Published:** 2025-08-29

**Authors:** Miloš Grujić, Stanislav Volchenkov, Aidos Akhmetali, Marija Živković Radojević, Neda Milosavljević, Katarina Janković, Katarina Krasić, Milica Mihajlović, Mohamed Shelan, Luca Nicosia, Mladen Marinković

**Affiliations:** 1 Center for Radiation Oncology, University Clinical Center Kragujevac, 34000 Kragujevac, Serbia; grujicmilos10@gmail.com (M.G.); makizivkovicmarija@gmail.com (M.Ž.R.); neda.milosavljevic@yahoo.com (N.M.); k.jankovic1991@gmail.com (K.J.); katarinakrasic@yahoo.com (K.K.); 2 Department of Clinical Oncology, Faculty of Medical Sciences, University of Kragujevac, 34000 Kragujevac, Serbia; 3 National Medical Research Center of Oncology N.N. Petrov, 197758 St. Petersburg, Russia; stanislav.volchenkov@yahoo.com; 4 Outpatient Care Department, Hematology Center LLP, Karaganda Medical University, Karaganda 100000, Kazakhstan; akhmetali.md@gmail.com; 5 Clinic for Oncology, University Clinical Center Nis, 18000 Nis, Serbia; dr.milicaradic@gmail.com; 6 Faculty of Medicine, University of Nis, 18000 Nis, Serbia; 7 Department of Radiation Oncology, Inselspital Bern, University of Bern, 3010 Bern, Switzerland; mohamed.shelan@insel.ch; 8 Advanced Radiation Oncology Department, IRCCS Sacro Cuore Don Calabria Hospital, Cancer Care Center, 37034 Verona, Italy; lucanicosia.rg@gmail.com; 9 Clinic for Radiation Oncology, Institute for Oncology and Radiology of Serbia, 11000 Belgrade, Serbia; 10 Faculty of Medicine, University of Belgrade, 11000 Belgrade, Serbia

**Keywords:** ultra-low-dose radiotherapy, ultra-low-dose radiation, ocular adnexal lymphoma, indolent lymphoma, local control, toxicity, systematic review

## Abstract

**Background/Objectives**: Ultra-low-dose radiotherapy (ULD-RT), an ultra-low-dose regimen delivering 4 Gy in two fractions, has emerged as a promising treatment for indolent ocular adnexal lymphoma (OAL), offering disease control with minimal toxicity. However, the clinical outcomes and safety profile of ULD-RT remain inconsistently reported across studies. **Methods**: We conducted a systematic review of peer-reviewed studies evaluating ULD-RT in patients with OAL, including prospective, retrospective, and comparative cohorts published between 2000 and 2025. A comprehensive search was performed in PubMed, Embase, and Scopus. Eligible studies reported clinical outcomes (e.g., response rates, local control, progression-free survival) and safety data (acute and late toxicities). Methodological quality was assessed using the Newcastle-Ottawa Scale (NOS). **Results**: Ten studies comprising 591 patients met the inclusion criteria. The overall response rate to ULD-RT ranged from 88% to 100%, with complete response rates of 50% to 95%. Local control rates ranged from 63% to 100%, and 2-year progression-free survival exceeded 85% in most studies. Importantly, no grade ≥ 3 toxicities were observed following the regimen of 4 Gy in two fractions. Acute toxicities occurred in up to 42% of patients, typically grade 1 dry eye or conjunctivitis. Late toxicities were uncommon (16–33%), with cataracts rarely requiring surgery. All included studies were rated as good quality (NOS score ≥ 7), indicating low risk of bias. **Conclusions**: ULD-RT is a highly effective and safe treatment modality for OAL, providing excellent local control with a favorable toxicity profile. These findings support the adoption of ULD-RT as a first-line radiotherapy strategy for indolent OAL. Future prospective trials with longer follow-up and standardized toxicity reporting are warranted to confirm and refine its clinical role.

## Simple Summary

This systematic review was conducted to address inconsistencies in the reported clinical outcomes and safety profile of ultra-low-dose radiotherapy (ULD-RT), an ultra-low-dose regimen for indolent ocular adnexal lymphoma (OAL). The aim was to evaluate the effectiveness and toxicity of ULD-RT by synthesizing data from published studies. High response rates, favorable local control, and minimal severe toxicities were identified across ten studies including 591 patients. These findings support the use of ULD-RT as a safe and effective first-line radiotherapy strategy for indolent OAL. The results may contribute to improved clinical decision-making and encourage future prospective studies with standardized outcome reporting to further define ULD-RT’s role in OAL treatment.

## 1. Introduction

According to data from 2022, non-Hodgkin lymphoma (NHL) ranks tenth globally in incidence and eleventh in mortality [1]. Ocular adnexal lymphoma (OAL) is a rare but clinically significant form of NHL that affects the orbit, conjunctiva, eyelids, and lacrimal glands, with extranodal marginal zone lymphoma of mucosa-associated lymphoid tissue (MALT) being the most common subtype [2]. While OAL is typically indolent, its anatomical location near critical visual structures presents unique treatment challenges, requiring a therapeutic approach that ensures high oncologic control while minimizing long-term ocular toxicity [3].

Historically, radiotherapy has been the cornerstone of OAL management, with early studies demonstrating excellent local control rates [4,5]. Initial radiation therapy techniques used orthovoltage X-rays, but these were associated with ocular toxicity, including keratitis, retinopathy, and cataract formation, due to poor dose conformity [6]. The transition to megavoltage linear accelerators and three-dimensional conformal radiation therapy (3D-CRT) significantly improved dose precision and toxicity profiles, leading to better clinical outcomes and safety profiles [7,8]. Most patients receive 20 to 40 Gy in 2 Gy fractions, whereas those with OAL have historically been treated with moderate to high doses (25–54 Gy), achieving outstanding local control rates of 90–100% [9].

In recent years, radiotherapy de-escalation strategies have gained prominence, particularly in indolent B-cell lymphomas, leading to the development of ultra-low-dose radiotherapy (ULD-RT), typically delivering 2 Gy × two fractions [10,11]. The aim of treatment modifications is to achieve equal local control while reducing the incidence of complications. Several studies have shown that ULD-RT achieves high disease control rates while significantly reducing late toxicities, making it the preferred radiotherapy option for localized OAL [12,13]. Additionally, ULD-RT has demonstrated notable cost-effectiveness, which may be particularly beneficial for reducing waiting lists and optimizing treatment access in healthcare systems with limited resources.

A variation of ULD-RT, referred to as boom-boom radiotherapy (BBRT), follows the same ultra-low-dose regimen (4 Gy in two fractions) and is simply an alternative name for ULD-RT used in select case reports and institutional protocols [14,15].

Proton beam therapy (PBT) has also emerged as a valid therapeutic option for OAL, offering highly conformal dose distribution with steep dose fall-off, thereby sparing adjacent critical ocular structures and potentially reducing the risk of late toxicity. Clinical series have reported excellent local control rates with PBT, comparable to conventional pho-ton techniques, particularly in cases where tumors are adjacent to radiosensitive structures such as the lens or optic nerve [16].

Several narrative reviews have summarized radiotherapy for OAL, generally underscoring conventional fractionation (20–30 Gy) with lens-sparing techniques as an effective standard for localized disease and outlining the broader therapeutic landscape [16,17]. More recently, dose and volume de-escalation strategies have been reviewed, suggesting that lower doses and smaller fields may maintain excellent local control while reducing toxicity [18]. In contrast to these broader reviews, the present article provides a focused, systematic synthesis of ultra-low-dose radiotherapy (4 Gy in two fractions, ULD-RT) for OAL, incorporating the latest prospective/response-adapted data and standardized toxicity binning, to clarify efficacy, safety, and practical use in contemporary practice.

The objective of this systematic review is to define the clinical outcomes and safety of ULD-RT in OAL, consolidating available evidence to improve treatment standardization and reporting. Given the widespread use of 4 Gy ULD-RT for OAL, this review aims to ensure the effective application and consistency of ULD-RT in clinical practice.

## 2. Materials and Methods

### 2.1. Criteria for Inclusion and Exclusion

Studies were selected based on predefined eligibility criteria to ensure methodological rigor and relevance.

The inclusion criteria encompassed studies involving patients diagnosed with ocular adnexal lymphoma (OAL), including mucosa-associated lymphoid tissue (MALT) lymphoma, follicular lymphoma, diffuse large B-cell lymphoma (DLBCL), and other subtypes affecting the eye, orbit, eyelid, conjunctiva, or lacrimal gland. Studies were required to evaluate the use of ultra-low dose radiotherapy (4 Gy in two fractions) for OAL, while comparative studies were also included if ULD-RT was one of the treatment arms. Eligible studies reported clinical outcomes such as local control rates, overall survival (OS), progression-free survival (PFS), response rates (complete response, partial response, stable disease, progressive disease), and recurrence rates. Safety outcomes, including ocular toxicity (such as dry eye, cataract, and retinopathy), systemic toxicity, and preservation of visual function, were also considered.

The study design was limited to randomized controlled trials (RCTs), prospective and retrospective cohort studies, case-control studies, and large case series with more than ten patients. Only full-text articles published in peer-reviewed journals were considered. Studies published in English or those with an available English translation were included. As an inclusion criterion, the publication timeframe was defined as 2000–2025.

Exclusion criteria were applied to ensure the relevance of the included studies. Case reports with fewer than ten patients, review articles, systematic reviews, meta-analyses, editorials, letters to the editor, abstract-only items, and commentaries were excluded. Studies published only as conference abstracts without sufficient data, as well as in vitro and animal studies, were not considered. Studies that did not evaluate ULD-RT or that focused solely on standard-dose or high-dose radiotherapy without a ULD-RT treatment arm were excluded, as well as studies with mixed head-and-neck series where orbital outcomes were not separable. Furthermore, studies that did not report relevant clinical or safety outcomes, as well as those with incomplete or unavailable full-text access, were not included in the analysis. Given the expected heterogeneity in design, cohorts, technique reporting, and follow-up, a descriptive synthesis was prespecified, with toxicity summarized using Common Terminology Criteria for Adverse Events (CTCAE) v5.0 grade bins when available.

### 2.2. Sources of Information

To identify relevant studies, a systematic search was conducted across three major databases: PubMed, Scopus, and Embase. PubMed was searched on 17 January 2025, followed by Scopus on 22 January 2025. Additionally, Embase was searched on 27 January 2025, to ensure comprehensive coverage of the available literature. These databases were selected due to their extensive indexing of biomedical and clinical research, ensuring the inclusion of high-quality peer-reviewed studies relevant to the topic. The review protocol was officially registered on the Open Science Framework under the identifier osf.io/djxfb.

### 2.3. Strategy for Literature Search

A structured search strategy was employed to identify relevant studies. This strategy was designed to capture studies investigating the use of ultra-low dose radiotherapy (4 Gy in two fractions) for ocular adnexal lymphoma while accounting for variations in terminology across different sources. The search was conducted using a combination of medical subject headings (MeSHs), relevant keywords, and Boolean operators to ensure comprehensive retrieval of pertinent literature. The following search string was applied across all selected databases:

“radiotherapy” AND (“boom boom” OR “low dose” OR “ultra-low” OR “4 Gy” OR “4 Gray” OR “two fraction”) AND lymphoma AND (ocul* OR adnex* OR orbit* OR choroid* OR eye)”

An asterisk (*) indicates truncation/wildcard to capture word variants (e.g., ocul* → ocular/oculoplastic), ensuring a broad and inclusive search.

### 2.4. Process for Selecting Studies

The first step in the process was deduplication. All retrieved references were imported into Zotero (version 7.0.11, available online: https://www.zotero.org, accessed on 29 January 2025) and Mendeley (version 1.19.8, available online: https://www.mendeley.com, accessed on 29 January 2025) for automatic deduplication, removing duplicate records before screening began. In the next step, two independent reviewers (M.G. and Ml.Ma.) screened the titles and abstracts to include only original articles. After this, full-text versions of the selected original articles were retrieved. These articles were then screened at the full-text level to ensure they met the inclusion and exclusion criteria. This final phase prioritized identifying studies that explicitly reported relevant interventions and outcomes. Any discrepancies in the selection process were resolved through discussion or, if necessary, by consulting a third reviewer (S.V.).

This systematic review follows Preferred Reporting Items for Systematic Reviews and Meta-Analyses (PRISMA) guidelines, ensuring rigorous methodology for literature selection, data extraction, and analysis [19].

### 2.5. Extraction of Data and Variables

Data extraction was performed independently by two reviewers (M.G. and Ml.Ma.) using a standardized form to maintain accuracy and consistency. Extracted information included study characteristics (first author, publication year, country, study design, and sample size), patient demographics (age and gender), and disease-related details (histology, affected anatomical site, laterality). Treatment-specific data encompassed ultra-low-dose radiotherapy parameters (dose, fractionation, and treatment arm inclusion), RT technique descriptors (modality and technique, beam energy, field arrangement, immobilization, lens shielding, bolus, planning approach, image guidance, and laterality strategy), as well as information related to prior therapy. Reported outcomes included clinical measures such as overall response rate (ORR), complete response (CR) rate, local control rate (LCR), OS, PFS, and safety outcomes, including acute and late toxicity. Discrepancies between reviewers were resolved by consensus discussion.

Acute and late toxicities were abstracted as reported in each study and harmonized to CTCAE v5.0 terminology and grade bins (0, 1–2, ≥3) when authors reported CTCAE grades or provided explicit grading. When CTCAE was not specified and only descriptive terms were available (e.g., “mild dry eye,” “cataract requiring surgery”), events were mapped to the closest CTCAE v5.0 term and grade bin and are denoted as author-reported, CTCAE-equivalent. No patient-level re-grading was performed. In response-adapted protocols, late events occurring after planned dose escalation (e.g., to 20–30 Gy) were attributed to the escalated dose and not to the initial 4 Gy.

### 2.6. Data Synthesis and Grouping of Study Outcomes

Data synthesis was conducted using a structured narrative approach. Studies were grouped based on clinical endpoints and reported outcomes. These included study design and characteristics, patient demographics, treatment protocols, key clinical outcomes (such as ORR, CR, LCR, OS, and PFS), and reported acute and late toxicities. Because only two studies [20,21] provided ULD-RT vs. MDRT comparisons, and they reported different endpoints, landmarks, and patient populations (site-specific orbital vs. pooled head-and-neck lymphomas), statistical meta-regression was not feasible, in accordance with the Synthesis Without Meta-analysis (SWiM) in Systematic Reviews guideline [22]. Instead, findings were summarized descriptively and presented in comparative tables. Trends and similarities across studies were evaluated qualitatively to identify consistent outcome patterns.

### 2.7. Assessment of Study Quality

The methodological quality and risk of bias of the included studies were independently assessed by two reviewers (M.G. and Ml.Ma.) using the Newcastle-Ottawa Scale (NOS) [23,24], a validated tool for evaluating the quality of non-randomized studies. This scale evaluates three domains: selection of study participants (maximum four stars), comparability of study groups (maximum two stars), and outcome assessment (maximum three stars), with a total maximum score of nine stars per study.

The reviewers performed their assessments independently, and any discrepancies were resolved through consensus discussion. No automation tools were used in the assessment process.

Given the descriptive nature of this review (no quantitative pooling), the small number of eligible studies, and the heterogeneity in endpoints and follow-up landmarks, formal small-study/publication-bias diagnostics (e.g., funnel plots, Egger’s/Begg’s tests) were not undertaken, because they are unlikely to be informative. To mitigate bias, three databases were searched, the reference lists of eligible articles were screened, and studies were included irrespective of outcome direction. The inclusion was limited to full-text original investigations. Case series with fewer than ten patients and abstract-only reports were excluded to minimize duplication and unstable estimates.

### 2.8. Dose Notation and Conventions

The radiotherapy dose was reported as “total dose (Gy) in N fractions (dose per fraction)”. For example, “4 Gy in two fractions (2 Gy per fraction)” is equivalent to “2 Gy × 2” or “4 Gy/2 fr”. Similarly, “24 Gy in 12 fractions (2 Gy per fraction)” may also appear as “24 Gy/12 fr” or “2 Gy × 12”. Phrases such as “20–40 Gy in 2 Gy fractions” denote 10–20 sessions of 2 Gy each to reach the total dose. (Abbreviation: fr = fractions).

## 3. Results

**Figure 1:**
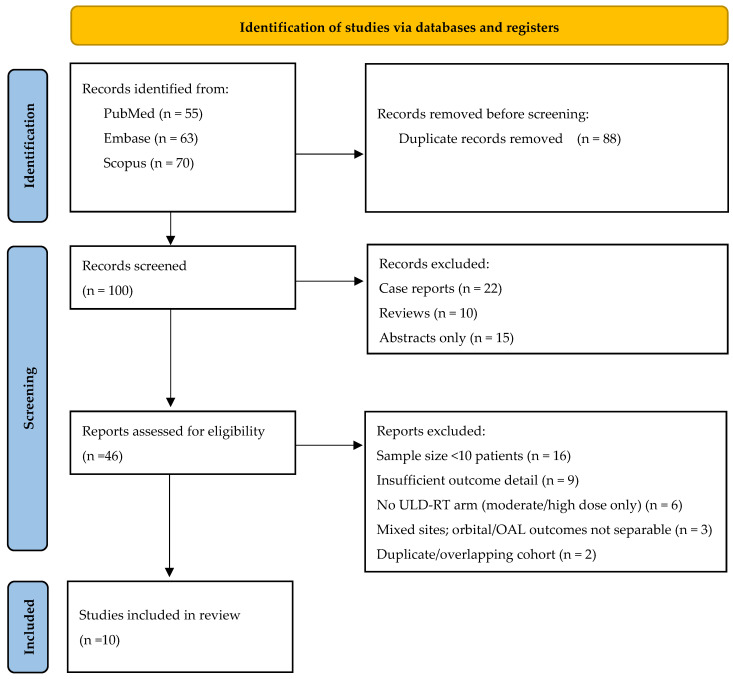
Flow chart diagram.

Of a total of 188 articles identified across three databases, 88 were duplicates, and 54 were excluded to focus on original articles. After applying the inclusion and exclusion criteria, ten articles were selected. The diagram of the selection process is presented in Figure 1. Of the selected articles, 80% were retrospective studies, and none were randomized controlled trials. The included studies represented cohorts from the USA, Australia, Asia, and Europe, covering four continents. The number of patients in the selected studies ranged from 14 to 266 (Table 1). All included studies scored between 7 and 8 on the Newcastle-Ottawa Scale (Supplementary Table S1), indicating low risk of bias and high methodological quality. The cohorts were generally well-defined and representative, outcome assessments were robust and consistent, and follow-up durations were appropriate. The primary limitation noted in some studies was insufficient adjustment for potential confounders in the comparability domain [20,21].

As part of Table 2, patient and disease characteristics, along with treatment protocols, are presented. The age range in the majority of studies covered patients from their twenties to their nineties. In all studies, females were more frequently represented, or the distribution was equal, except in the study by Manta et al. [29], where three-quarters of the patients were male. The most common histological type was MALT lymphoma, although in studies where MALT lymphoma was not included [12,20,21,30], the most common types were marginal zone lymphoma (MZL) and follicular lymphoma (FL) [12,20,21,30]. The conjunctiva and orbit were the most common sites. Around 30% of patients (12–42%) had bilateral disease.

In all studies, patients were treated using a regimen of 4 Gy in two fractions (ULD-RT). Radiotherapy techniques were variably reported across studies. Where specified, modality/technique (3D-CRT/IMRT/VMAT/electrons/protons), beam energy, field arrangement, immobilization, and lens shielding were recorded. A consolidated per-study summary is provided in Supplementary Table S2. Chelius et al. and Baron et al. presented data on moderate-dose RT (more than 4 Gy) compared to the group treated with the ULD-RT [20,21].

The included studies varied regarding prior treatment, with 50% of the studies including only naïve patients who had not received treatment before evaluation of the RT response. Among studies that reported prior treatment, the proportion with any prior systemic therapy ranged from 0% to approximately 45%; several cohorts also permitted or reported concurrent/sequence rituximab. No study provided outcomes stratified by prior systemic therapy (Table 2).

Table 3 presents the study endpoints. Ultra-low-dose radiotherapy (ULD-RT, 4 Gy in two fractions) demonstrated high response rates across all studies. As the studies reported outcomes at different time points, the results are presented accordingly, and direct cross-study comparison should be interpreted with caution. The overall response rate (ORR) ranged from 88% to 100%, with CR rates between 50% and 95%, depending on the study population [21,29]. The local control (LC) rates ranged from 63% to 100% depending on the follow-up period, showing that ULD-RT provided durable disease control similar to medium-dose radiotherapy (MDRT) [10,12,20,21]. Minimal disease progression was observed, with local relapse rates ranging from 2% to 5% across studies [21,30]. The 2-year PFS rates ranged from 85% to 100%, indicating a low likelihood of relapse [25]. Overall survival (OS) at 2 years ranged from 98% to 100%, demonstrating that patients receiving ULD-RT had excellent long-term survival prospects [10,25].

Among the included studies, only two cohorts directly contrasted ULD-RT (4 Gy in two fractions) with moderate-dose regimens (typically 20–30 Gy): Baron et al. (indolent orbital adnexal lymphomas) and Chelius et al. (indolent head-and-neck lymphomas, including an orbital subset) [20,21]. In Baron’s orbital-only analysis, the overall response was high across both dose groups, and local/orbital control at the 2-year landmark was comparable between ULD-RT and MDRT [21]. Because endpoints, landmarks, and case mix differ between these two studies, a formal dose–response meta-regression was not performed.

Table 4 and Table 5 present number of patients experiencing acute or late toxicity. Ultra-low-dose radiotherapy (ULD-RT) was associated with significantly lower acute toxicity compared to MDRT in studies that included a comparison group [20,21]. Three of the included studies of patients treated with the ULD-RT regimen reported no acute toxicities, while in others 6–42% of patients experienced mild, grade 1 toxicities (e.g., dry eye, conjunctivitis, periorbital edema). In contrast, acute toxicity rates were much higher in the MDRT group, reaching up to 96%, with cataract formation and conjunctivitis being the most common toxicities [20]. Regarding late effects, ULD-RT was associated with minimal long-term side effects, particularly compared to MDRT. Late toxicity rates were significantly lower with ULD-RT (16–33%) compared to MDRT (42–71%) [20,21]. Mild dry eye was the most frequently reported late effect. Cataract formation was rare in the ULD-RT groups, whereas in the MDRT groups, the incidence of cataracts requiring surgery ranged from 4% to 28% [20,21]. Notably, no grade 3 or higher toxicities were observed in ULD-RT-treated patients across multiple studies [10,21]. The sole grade 2 cataract occurred in a response-adapted cohort after escalation to 24 Gy, whereas cataracts requiring surgery were reported only in MDRT groups [10]. Toxicity harmonization according to CTCAE v5.0 is presented in Supplementary Table S3.

Several cohorts reported bilateral orbital involvement, typically ranging from 10% to 30% of patients. For example, bilateral disease was treated in Fasola et al., with one patient developing bilateral cataracts at 7 years that was considered unlikely to be radiation-related [30]. Shelukar et al. included bilateral cases (12%) and observed low oncologist-graded toxicity (6%) but higher rates of mild findings on ophthalmologic assessment (33.3%) at the cohort level [26]. In the 2-year series by Manta et al., 14% underwent bilateral treatment, with overall late toxicity limited to mild dry eye in 14% [29]. The prospective study by Yang et al. reported no acute or chronic toxic effects at the cohort level [25]. No study provided laterality-stratified toxicity, and a pooled comparison of bilateral vs. unilateral outcomes was therefore not feasible.

## 4. Discussion

This systematic review assessed the clinical outcomes and safety of ultra-low-dose radiotherapy (ULD-RT, 4 Gy in two fractions) in patients with OAL. The findings confirm that ULD-RT achieves high ORR (88–100%), with CR rates ranging from 50% to 95%. Additionally, ULD-RT provided durable disease control, with an LCR of 63–100% and a low LLR (2–5%). Progression-free survival at 2 years ranged from 85% to 100%, while OS exceeded 98%, indicating excellent long-term outcomes. Importantly, ULD-RT was associated with significantly lower acute and late toxicities compared to MDRT. While some patients experienced mild, grade 1 acute toxicities (6–42%), three studies reported no acute toxicities at all. Late toxicities were minimal, with mild dry eye being the most common, and the incidence of cataracts requiring surgery remained low compared to MDRT. No grade 3 or higher toxicities were observed in ULD-RT-treated patients across multiple studies. These findings support ULD-RT as a safe and effective treatment approach for OAL, reinforcing its role in clinical practice. This could be particularly relevant in elderly or frail patients, in whom toxicity may represent a critical issue. Although many included patients were elderly, few studies stratified toxicity or efficacy outcomes by age or performance status (PS). This represents a gap in understanding the suitability of ULD-RT in frail populations.

The clinical effectiveness of ULD-RT in indolent B-cell lymphomas likely stems from their inherently high radiosensitivity. Indeed, ultra-low-dose RT regimens (2 × 2 Gy) produce approximately 90% overall response rates across diverse indolent lymphoma subtypes, including orbital presentations [31]. Preclinical investigations further reveal that even low-dose radiation robustly activates the p53 pathway, inducing apoptosis in lymphoma cells [32]. Moreover, low-dose RT may trigger immune-mediated tumor control, and studies indicate enhanced dendritic cell activation and systemic anti-lymphoma responses following radiation [33]. While further mechanistic research specific to OAL is needed, these data support a plausible biological framework for the efficacy of ULD-RT beyond direct tumor cytotoxicity. In ocular adnexal lymphomas, these mechanisms may be further shaped by the unique orbital microenvironment [31]. Conjunctiva-associated lymphoid tissue (CALT) and local immunoregulatory mediators (e.g., TGF-β2, Fas-ligand) may amplify the immune-modulatory effects of ultra-low doses [34]. Moreover, the concept of ocular immune privilege, combined with the relatively high radiation sensitivity of lymphoid tissue and the wide therapeutic margin of orbital organs at risk, may explain how ultra-low-dose regimens achieve effective disease control with minimal collateral toxicity [35].

From a health system perspective, ultra-low-dose radiotherapy (ULD-RT; 4 Gy in two fractions) is expected to be less resource-intensive than conventionally fractionated radiotherapy (CFR, typically 20–30 fractions for indolent OAL). The drivers are primarily fraction-linked, including fewer treatment visits, less machine time, fewer on-treatment visits, and fewer image-guidance events. Planning and quality assurance requirements are comparable when simple conformal techniques are used, and planning effort is similar between ULD-RT and CFR, but the overall delivery burden remains substantially lower with two fractions. For patients and caregivers, ULD-RT reduces travel and time costs by an order of magnitude relative to CFR (2 vs. around 20–30 visits). Downstream costs may also differ. Prospective economic evaluations, ideally alongside clinical cohorts, should capture payer and societal perspectives, incorporate quality-adjusted outcomes, and report budget-impact implications for throughput and waiting lists.

Beyond the core studies included in our systematic review, several additional publications provide further context and support for ULD-RT. Yang et al. presented three consecutive cases of choroidal lymphoma treated with 4 Gy in two fractions, demonstrating complete response in all patients without toxicity [14]. These findings highlight the efficacy and tolerability of ULD-RT in delicate intraocular settings, further expanding its potential applicability beyond classic adnexal presentations. Flanagan et al. and Astafurov et al. also reported promising responses in uncommon scenarios such as choroidal involvement and post-transplant lymphoproliferative disorder [15,36].

Importantly, some studies excluded from the systematic review due to design or population differences—such as case reports, short follow-up, or small sample sizes—nonetheless reinforce the overall findings. For example, case series involving IgG4-related ophthalmic disease and choroidal lymphoma confirm both the feasibility and efficacy of ULD-RT, even in atypical anatomical presentations [11]. Reports on meibomian gland dysfunction, vitreoretinal lymphoma, and radiation therapy for conjunctival marginal zone lymphoma suggest broader ophthalmologic implications and encourage more detailed toxicity profiling using standardized ophthalmologic assessments [37,38,39].

Within the broader movement to de-escalate orbital radiotherapy, ultra-low-dose radiotherapy (ULD-RT; 4 Gy in two fractions) represents the best-studied ultra-low-dose approach for indolent OAL. Multiple retrospective and prospective cohorts, including response-adapted protocols in which non-CR lesions are escalated to 24–30 Gy, show high response and excellent short-term local control with substantially lower ocular toxicity compared with historical moderate-dose regimens. By contrast, single-fraction 2 Gy (2 Gy × 1) has very limited OAL-specific evidence and has primarily been described as an exploratory or anecdotal practice, rather than as a validated definitive strategy for the orbit [40]. Accordingly, our findings support ULD-RT as the current de-escalation standard with the strongest evidentiary base in OAL, while highlighting the need for prospective, ophthalmology-led studies directly comparing 2 Gy × 1, 2 Gy × 2, and conventional doses using harmonized endpoints.

Radiotherapy technique represents an additional variable with potential impact on the safety outcomes reported in ULD-RT studies. While the majority of studies included in our systematic review did not stratify toxicity outcomes by treatment technique, it is important to recognize that highly conformal techniques such as intensity-modulated radiotherapy (IMRT) may contribute to minimizing radiation exposure to critical ocular structures. Although our pooled data do not provide technique-specific comparisons, this general principle may be relevant when interpreting safety considerations in ultra-low-dose regimens. In this context, data from external sources not included in our systematic review offer valuable insights. For example, a large retrospective cohort by Rehn et al. demonstrated a reduction in grade 2 late toxicity from 33% to 9% when IMRT was employed compared to conventional techniques [18]. Although this study did not meet our inclusion criteria, it supports the hypothesis that modern radiotherapy techniques may enhance the safety profile of ULD-RT. As lesion location often dictates the use of electrons (e.g., conjunctival disease) versus photons (e.g., intraorbital disease), future studies should aim to control for technical variation to more accurately isolate the impact of technique on treatment-related toxicity.

The role of systemic therapy in the analyzed studies warrants consideration when interpreting ULD-RT outcomes. In certain cohorts, such as those included in Baron et al. and Fasola et al., a subset of patients received rituximab or prior chemotherapy, potentially influencing response rates and progression-free survival independent of the radiation dose [21,30]. Conversely, studies such as Yang et al. strictly excluded patients with previous systemic therapy, thereby providing a more isolated evaluation of ULD-RT efficacy [25]. The heterogeneity in systemic treatment exposure across studies represents a potential confounder, particularly in retrospective analyses where treatment allocation was not standardized. Nonetheless, the majority of ULD-RT data reflect outcomes in early-stage, indolent lymphomas, where systemic therapy is not routinely indicated. As such, the favorable outcomes associated with ULD-RT in these contexts likely reflect its intrinsic therapeutic value. While several cohorts enrolled previously treated patients or permitted concurrent rituximab, the lack of stratified outcomes by prior systemic therapy precluded comparative analysis in this review. Future reports should predefine prior-therapy categories (e.g., treatment-naïve vs. relapsed/refractory) and present response/toxicity stratified accordingly to clarify the generalizability of ULD-RT across treatment histories.

In indolent OAL, non-cancer mortality can compete with local events, particularly in older or frail patients; however, the included studies did not report age- or PS-stratified LC/LPFS/ORR, limiting risk-adjusted inference. Future prospective cohorts should pre-specify age/PS strata and use competing-risk methods with standardized 2- and 5-year endpoints.

Additional real-world evidence strengthens the position of ULD-RT in the management of ocular adnexal lymphoma. A retrospective analysis by Chelius et al. demonstrated significantly lower early and late toxicity rates across all treatment sites—including the orbit—when using 4 Gy versus standard-dose regimens [20]. Importantly, no high-grade toxicities occurred in the 4 Gy group, while 17 cases of grade 3 cataracts were documented in the >24 Gy group, highlighting the favorable safety profile of ULD-RT [20]. Similarly, König et al. reported excellent local control and complete responses in all patients treated with ULD-RT, though distant relapse occurred in advanced-stage patients, underscoring the importance of appropriate staging and long-term follow-up [12]. Shelukar et al. further revealed that ophthalmologist-led evaluations detected more subtle toxicities than oncologist assessments, advocating for comprehensive ocular surveillance in future studies [26]. Collectively, these findings support the integration of ULD-RT into routine practice for early-stage OAL, particularly when treatment aims to minimize toxicity while preserving local control. Because the included studies provided limited detail on how ophthalmic evaluations were performed, we refrained from imposing instrument-level standards beyond CTCAE grade-bin harmonization. For future research, we recommend predefining the assessor (ophthalmology-led or multidisciplinary), a concise set of core domains (visual function, anterior segment/ocular surface, lens status, intraocular pressure, posterior segment, ocular motility/lids/lacrimal system), the unit of analysis (patient vs. eye), and common timepoints (baseline, early follow-up, 12 months, and annually), with adverse events graded by CTCAE v5.0.

In comparison, Goda et al. reported their institutional experience with 25–30 Gy RT in 89 patients, noting a 45% rate of late toxicity—primarily cataracts—with a median time to formation of 3.6 years [41]. Importantly, grade 3 cataract incidence was reduced from 41% to 15% with lens shielding. While acute effects were mild and self-limiting, these findings emphasize the cumulative burden of late effects with moderate doses, especially in long-surviving patients. This further supports the use of ULD-RT in selected cases to reduce long-term ocular morbidity while preserving efficacy in indolent lymphomas. It offers a low-toxicity, cost-effective alternative with minimal resource utilization, which is particularly valuable in healthcare systems with limited access to advanced radiation technologies. Studies by König et al. and Chelius et al. emphasize that ULD-RT does not preclude subsequent full-dose re-irradiation in cases of relapse, further supporting its use as an initial conservative strategy [12,20]. Furthermore, the delayed complete responses observed in multiple studies justify extended observation before treatment intensification to reduce overtreatment risks. Importantly, many radiation-induced ocular sequelae, including cataract formation and some manifestations of radiation retinopathy, have a latency exceeding 5 years. Within the included literature, only one cohort (König et al.) reports 5-year outcomes, while most of the others have a median follow-up of ≤2–3 years [12]. As a result, our pooled description of late toxicity should be interpreted as a minimum estimate. Long-term (≥5-year) ophthalmology-led surveillance with standardized grading is essential to accurately characterize late effects.

Several limitations should be considered when interpreting the findings from this systematic review. First, the majority of the studies included were retrospective in nature, which inherently limits the ability to establish causality and introduces potential biases. We deliberately restricted inclusion to ULD-RT (4 Gy in two fractions) to maintain a focused question. Adding conventional-dose RT series without a ULD-RT arm would enlarge the dataset but materially increase heterogeneity and reduce ULD-RT-specific interpretability. Therefore, such studies are cited for context but were not pooled with the ULD-RT evidence. Additionally, most of the studies had small sample sizes, with patient numbers ranging from 14 to 47, which may affect the generalizability and statistical power of the results. Furthermore, some studies included patients who had previously received treatment, making it challenging to isolate the specific effect of ULD-RT. Another limitation is the relatively short follow-up periods in the majority of the studies, which limits our understanding of the long-term benefits and safety of ULD-RT. The standardized reporting of toxicities was also inconsistent across studies, which may explain the variation in reported incidence rates. While the evidence suggests that ULD-RT is effective in treating OAL, stronger evidence is needed to confirm these findings. Publication and reporting bias may remain despite our search strategy. Retrospective designs, selective outcome reporting, and preferential publication of favorable results could overestimate control and underestimate toxicity. Excluding small (<10 patients) and abstract-only series reduces imprecision and duplication but may miss negative or null reports. Prospective, registered, ophthalmology-led studies using standardized endpoints (e.g., 2- and 5-year LC/LPFS) and CTCAE v5.0 toxicity grading are needed to mitigate these biases.

While our synthesis supports ULD-RT as an effective and well-tolerated option for indolent OAL, prospective evidence is needed to strengthen causal inference and refine patient selection. Pragmatic, multicenter prospective cohorts or, where feasible, randomized non-inferiority designs against MDRT with preregistered protocols and prespecified core endpoints (e.g., 2- and 5-year local control and local progression-free survival, time to next ocular/systemic therapy, ocular toxicity, and patient-reported vision-related quality of life) are needed. Standardized CTCAE v5.0 grading with clearly defined assessment responsibility, reporting of the unit of analysis, and fixed assessment timepoints will improve comparability. Studies should also plan follow-up beyond 5 years to quantify late effects, incorporate competing-risk methods for older/frail populations, and report outcomes stratified by laterality, prior systemic therapy, and age/performance status. Finally, response-adapted protocols should clearly attribute outcomes and toxicities to the initial versus escalated dose to enable valid cross-study comparisons.

## 5. Conclusions

Ultra-low-dose radiotherapy (ULD-RT, 4 Gy in two fractions) demonstrates high response rates (88–100%) and excellent long-term local control (63–100%), with low recurrence rates (2–5%). Additionally, ULD-RT significantly reduces both acute and late toxicities, including cataracts and severe dry eye syndrome, when compared to MDRT. Response-adapted approaches further optimize treatment by minimizing radiation exposure while maintaining excellent outcomes. Although current evidence strongly supports the use of ULD-RT as first-line therapy for indolent orbital lymphomas, larger prospective, ophthalmology-led trials are needed in the future to confirm these findings and establish the role of ULD-RT in standard clinical practice. Standardized reporting of toxicities and long-term follow-up are crucial to further strengthen the evidence base.

## Figures and Tables

**Table 1:** Characteristics of the included studies.

Author (Citation)	Year	Country	Study Design	Sample Size ^a^
Pinnix [10]	2024	United States	Clinical trial (phase 2, single-arm) and retrospective study	105 ^b^
Yang [25]	2022	China	Prospective exploratory study	16
Shelukar [26]	2022	United States	Retrospective case series	17
Pinnix [27]	2017	United States	Retrospective study	22
Park [28]	2022	Korea	Clinical trial (phase 2, single-arm)	14
Manta [29]	2024	Australia	Retrospective study	21
König [12]	2018	Germany	Retrospective study	47
Fasola [30]	2013	United States	Retrospective study	20
Chelius [20]	2021	United States	Retrospective study	266 ^c^
Baron [21]	2021	United States	Retrospective study	36

^a^ Number of patients; ^b^ 50 patients included in prospective arm and 55 patients included in retrospective study; ᶜ reports an indolent head-and-neck cohort (N = 266) that includes an orbital subset; for orbit-specific analyses in this review, the subset comprised N= 81 (4 Gy N = 12; >4 Gy N = 69).

**Table 2:** Patient and disease characteristics, with treatment protocols.

Author (Citation)	Age, Median (Range)	Gender (Female/Male)	Histology	Disease Location	Laterality	RT Dose	Prior Therapy
		Number of Patients (%)					
Pinnix–prospective cohort [10]	63 (29–88)	31 (62%)/19 (38%)	MALT ^a^: 32 (64%) FL ^b^: 12 (24%) Low-grade unspecified: 6 (12%)	Conjunctiva only: 16 (32%) Lacrimal gland only: 6 (12%) Soft tissues/muscles: 23 (46%) Multiple sites: 5 (10%)	Uni ^c^: 42 (84%) Bi ^d^: 8 (16%)	4 Gy/2 fr.	No systemic therapy within 4 weeks before radiotherapy; newly diagnosed: 36 (72%) Systemic treatment after radiotherapy (0.1–53.6 months): 10 (20%)
Pinnix–retrospective cohort [10]	63 (25–87)	26 (47%)/29 (53%)	MALT ^a^: 38 (69%) FL ^b^: 13 (24%) Low-grade unspecified: 4 (7%)	Conjunctiva only: 17 (31%) Lacrimal gland only: 7 (13%) Soft tissues/muscles: 16 (29%) Multiple sites: 15 (27%)	Uni ^c^: 39 (71%) Bi ^d^: 16 (29%)	4 Gy/2 fr.	No systemic therapy within 4 weeks before radiotherapy; newly diagnosed: 44 (80%)
Yang [25]	63 (23–86)	7 (44%)/9 (56%)	MALT ^a^: 11 (69%) FL ^b^: 2 (12%) Lymphoid hyperplasia: 3 (19%)	Conjunctiva: 5 (19%) Lacrimal gland: 2 (7%) Soft tissue: 11 (41%) Rectus: 4 (14%) Eyelid: 5 (19%)	Uni ^c^:11 (69%) Bi ^d^: 5 (31%)	4 Gy/2 fr.	No other therapy concomitantly with radiotherapy until evaluation of the response
Shelukar [26]	67 (24–80)	12 (71%)/5 (29%)	MALT ^a^: 5 (29%) FL ^b^: 4 (24%) MZL ^e^: 3 (18%) MCL ^f^: 1 (6%) Other low-grade: 4 (24%)	Conjunctiva: 4 (21%) Lacrimal gland: 4 (21%) Orbit: 8 (42%) Choroid: 2 (11%) Eyelid: 5 (5%)	Uni ^c^: 15 (88%) Bi ^d^: 2 (12%)	4 Gy/2 fr.	No previous treatment
Pinnix [27]	64.5 (25–88)	12 (55%)/10 (45%)	MALT ^a^: 14 (64%) FL ^b^: 5 (23%) MCL ^f^: 2 (9%) Low-grade unspecified: 1 (4%)	Conjunctiva: 6 (27%) Lacrimal gland: 6 (27%) Soft tissue: 9 (41%) Mixed: 1 (5%)	Uni ^c^: 16 (73%) Bi ^d^: 6 (27%)	4 Gy/2 fr.	Prior systemic treatment: 10 (45%) Prior orbital radiotherapy: 1 (5%)
Park [28]	60.5 (NA ^g^)	7 (50%)/7 (50%)	NA ^g^	Conjunctiva: 14 (82.3%) Retrobulbar: 1 (5.9%) Mixed: 2 (11.8%)	Uni ^c^:11 (78.6%) Bi ^d^: 3 (21.4%)	4 Gy/2 fr.	No previous radiotherapy in the orbit
Manta [29]	62 (33–85)	6 (29%)/15 (71%)	MZL ^e^: 14 (67%) FL ^b^: 5 (24%) MCL ^f^: 2 (10%)	Conjunctiva: 9 (43%) Orbit: 14 (67%) Lacrimal gland: 7 (33%) Extraocular muscles: 4 (19%) Eyelid/preseptal tissue: 4 (19%)	Uni ^c^: 18 (85.7%) Bi ^d^: 3 (14.3%)	4 Gy/2 fr.	No concomitantly chemotherapy; 5 (24%) had a history of prior treatment for ocular adnexal and/or systemic NHL ^i^
König [12]	64 (33–89)	29 (61.7%)/18 (38.3%)	FL ^b^: 27 (57.4%) MZL ^e^: 20 (42.6%)	Nodal disease: 8 lesions (16%) Extranodal disease: 42 lesions (84%): Orbit: 16 lesions (32%) Salivary glands: 7 lesions (14%) Skin: 15 lesions (30%) Others: 4 lesions (8%)	NA ^g^	4 Gy/2 fr.	Rituximab simultaneously: 13 (26%) Primary treatment: 30 (60%)
Fasola [30]	70 (38–88)	10 (50%)/10 (50%)	FL ^b^: 11 (55%) MZL ^e^: 8 (40%) MCL ^f^: 1 (5%)	Conjunctiva: 9 (33%) Lacrimal gland: 11 (41%) Retrobulbar: 2 (7%) Eyelid: 5 (19%)	Uni ^c^: 14 (70%) Bi ^d^: 6 (30%)	4 Gy/2 fr.	Prior chemotherapy: 6 (30%) Prior radiotherapy: 8 (40%)
Chelius [20]	61 (19–92)	126 (47.4%)/140 (52.6%)	FL ^b^: 117 (44.1%) MZL ^e^/MCL ^f^: 110 (41.4%) Cutaneous B-cell: 22 (8.3%) Small lymphocytic lymphoma/CLL ^h^: 17 (6.4%)	Orbit: 81 (30.5%)	NA ^g^	4 Gy/2 fr. (12/62 pts orbit)	Relapse or refractory disease: 29 (46.8%)
						>4 Gy (69/204 pts orbit)	Relapse or refractory disease: 32 (15.7%)
Baron [21]	64.5 (16–92)	23 (64%)/13 (36%)	MZL ^e^: 20 (56%) FL ^b^: 11 (30%) MCL ^f^: 5 (14%)	Conjunctiva: 7 (19%) Lacrimal gland: 13 (36%) Eyelid: 15 (42%) Other: 1 (3%)	Uni ^c^: 21 (58%) Bi ^d^: 15 (42%)	4 Gy/2 fr. (12 pts)	Concurrent/sequential Rituximab: 8 (22%)
						Median RT dose 24 Gy (21–36 Gy) (24 pts)	

^a^ MALT—mucosa-associated lymphoid tissue; ^b^ FL—follicular lymphoma; ^c^ Uni—unilateral; ^d^ Bi—bilateral; ^e^ MZL—marginal zone lymphoma; ^f^ MCL—mantle cell lymphoma; ^g^ NA—not available; ^h^ CLL—chronic lymphocytic leukemia; ^i^ NHL—non-Hodgkin lymphoma.

**Table 3:** Study endpoints and key outcomes.

Author (Citation)	Overall Response Rate (ORR) ^b^	Complete Response (CR ^d^)	Local Control Rate (LCR)					Progression-Free Survival (PFS)			Overall Survival (OS)		
	Number of Patients (%)		1-Year	2-Year	3-Year	4-Year	5-Year	1-Year	2-Year	5-Year	1-Year	2-Year	5-Year
Pinnix—prospective cohort [10]	NA ^a^	44 (88%)	NA ^a^	89%	NA ^a^	NA ^a^	NA ^a^	NA ^a^	NA ^a^	NA ^a^	NA ^a^	98%	NA ^a^
Pinnix—retrospective cohort [10]	NA ^a^	51 (93%)	NA ^a^	96%	NA ^a^	NA ^a^	NA ^a^	NA ^a^	NA ^a^	NA ^a^	NA ^a^	98%	NA ^a^
Yang [25]	14 (88%)	12 (75%)	85%	NA ^a^	NA ^a^	NA ^a^	NA ^a^	LPFS ^c^ 85% DPFS ^g^ 100%	NA ^a^	NA ^a^	100%	NA ^a^	NA ^a^
Shelukar [26]	15 (89%)	11 (65%)	NA ^a^	NA ^a^	100%	NA ^a^	94%	NA ^a^	NA ^a^	NA ^a^	NA ^a^	NA ^a^	NA ^a^
Pinnix [27]	22 (100%)	19 (86%)	NA ^a^	NA ^a^	NA ^a^	NA ^a^	NA ^a^	LPFS ^c^ 100% (for 19 patients who achieved CR ^d^)	LPFS ^c^ 75% (for 19 patients who achieved CR ^d^)	NA ^a^	NA ^a^	NA ^a^	NA ^a^
Park [28]	17/17 Ls ^e^ (100%)	11/17 Ls ^e^ (64%)	NA ^a^	91% ^f^	NA ^a^	NA ^a^	NA ^a^	NA ^a^	90% ^f^	NA ^a^	NA ^a^	100% ^f^	NA ^a^
Manta [29]	20 (95%)	20 (95%)	NA ^a^	100%	NA ^a^	NA ^a^	NA ^a^	NA ^a^	100%	NA ^a^	NA ^a^	100%	NA ^a^
König [12]	45/50 Ls ^e^ (90%)	43/50 Ls ^e^ (82%)	NA ^a^	91% ^c^	NA ^a^	NA ^a^	NA ^a^	NA ^a^	DPFS ^g^ 83%	DPFS ^g^ 83%	NA ^a^	96.6%	88.6%
Fasola [30]	26/27 Ls ^e^ (96%)	23/27 Ls ^e^ (85%)	NA ^a^	NA ^a^	NA ^a^	NA ^a^	NA ^a^	NA ^a^	LPFS ^c^ 100% RPFS ^h^ 96% ^i^ DPFS ^g^ 75%	NA ^a^	NA ^a^	NA ^a^	NA ^a^
Chelius—4 Gy cohort [20]	NA ^a^	45/62 (73%)	40/45 (89%) ^j,k^	NA ^a^	NA ^a^	NA ^a^	NA ^a^	NA ^a^	NA ^a^	NA ^a^	NA ^a^	NA ^a^	NA ^a^
Chelius—>4 Gy cohort [20]	NA ^a^	92/104 (89%)	84/92 (96%) ^j^	NA ^a^	NA ^a^	NA ^a^	NA ^a^	NA ^a^	NA ^a^	NA ^a^	NA ^a^	NA ^a^	NA ^a^
Baron—ULD-RT ^l^ [21]	100%	50%	NA ^a^	100%	NA ^a^	100%	NA ^a^	NA ^a^	NA ^a^	NA ^a^	NA ^a^	100%	NA ^a^
Baron—MDRT ^m^ [21]	88%	58%	NA ^a^	100%	NA ^a^	89%	NA ^a^	NA ^a^	NA ^a^	NA ^a^	NA ^a^	95%	NA ^a^

^a^ NA—not available; ^b^ ORR—overall response rate [patients who achieved either a complete response (CR) or partial response (PR)]; ^c^ LPFS—local progression-free survival; ^d^ CR—complete response; ^e^ Ls—lesions; ^f^ two patients with initial partial response (PR) underwent salvage treatment with a radiotherapy dose of 24 Gy in 12 fractions, and it is not possible to attribute the isolated effect to the initially administered dose of 4 Gy in two fractions; ^g^ DPFS—distant progression-free survival; ^h^ RPFS—regional progression-free survival; ^i^ within the cohort in which complete response (CR) was achieved; ^j^ in the subgroup of patients where complete response (CR) was achieved (as reported—median follow-up was 23 months (2–145) and 68 months (2–256) for 4 Gy and >4 Gy cohorts, respectively); ^k^ three patients were lost to follow-up; ^l^ ULD-RT—ultra-low-dose radiotherapy; ^m^ MDRT—medium-dose radiotherapy.

**Table 4:** Reported acute toxicities following radiotherapy.

Author (Citation)	Acute Toxicity			Most Common Type Number of Patients (%)
	Number of Patients (%)			
	Grade 1	Grade 2	Grade 3	
Pinnix—prospective cohort [10]	3 (6%)	1 (2%)	No toxicity	Dry eye 4 (8%)
Pinnix—retrospective cohort [10]	1 (1.8%)	No toxicity	No toxicity	HSV ^a^ keratitis 1 (1.8%)
Yang [25]	No toxicity	No toxicity	No toxicity	/
Shelukar [26]	Patients followed by oncologist:			NA
	1 (6%)	No toxicity	No toxicity	
	Patients followed by ophthalmologist:			
	5 (33.3%)	No toxicity	No toxicity	Dry eye 3 (20%), cataract 1 (6.7%), chorioretinal atrophy 1 (6.7%)
Pinnix [27]	1 (4.5%)	No toxicity	No toxicity	Dry eye 1 (4.5%)
Park [28]	1 (7%)	No toxicity	No toxicity	Dry eye 1 (7%)
Manta [29]	No toxicity	No toxicity	No toxicity	/
König [12]	No toxicity	No toxicity	No toxicity	/
Fasola [30]	6 (23%)	No toxicity	No toxicity	Dry eye 1 (4%), acute conjunctivitis 1 (4%), transient periorbital edema 4 (15%)
Chelius—ULD-RT ^b^ [20]	Orbit: 5/12 (41.7%)		No toxicity	Xerophthalmia 3 (25%) Visual changes 1 (8%) Dermatitis 1 (8%)
Chelius—MDRT ^c^ [20]	Orbit: 66/69 (95.7%)		No toxicity	Xerophthalmia 34 (49%) Conjunctivitis 20 (29%) Visual changes 17 (25%) Watering eye 21 (30%)
Baron—ULD-RT ^b^ [21]	ULD-RT ^b^: 6 (50%)		No toxicity	NA
Baron—MDRT ^c^ [21]	MDRT ^c^: 20 (83.3%)		No toxicity	NA

^a^ HSV—herpes simplex virus; ^b^ ULD-RT—ultra-low-dose radiotherapy; ^c^ MDRT—medium-dose radiotherapy; NA—not available.

**Table 5:** Reported late toxicities following radiotherapy.

Author (Citation)	Late Toxicity			Most Common Type Number of Patients (%)
	Number of Patients (%)			
	Grade 1	Grade 2	Grade 3	
Pinnix—prospective cohort [10]	No toxicity	No toxicity	No toxicity	/
Pinnix—retrospective cohort [10]	No toxicity	No toxicity *	No toxicity	/
Yang [25]	No toxicity	No toxicity	No toxicity	/
Shelukar [26]	No toxicity	No toxicity	No toxicity	/
Pinnix [27]	No toxicity	No toxicity	No toxicity	/
Park [28]	No toxicity	No toxicity	No toxicity	/
Manta [29]	3 (14%)		No toxicity	Mild dry eye 3 (14%)
König [12]	No toxicity	No toxicity	No toxicity	/
Fasola [30]	No toxicity	No toxicity	No toxicity	/
Chelius—ULD-RT ^a^ [20]	ULD-RT ^a^—orbit: 2/10 (20%)		No toxicity	Xerophthalmia 1 (10%) Conjunctivitis 1 (10%) Dermatitis 1 (10%)
Chelius—MDRT ^b^ [20]	MDRT ^b^orbit: 48/68 (70.6%)		No toxicity	Xerophthalmia 28 (41%) Cataracts 19 (28%) Visual changes 18 (27%) Watering eye 11 (16%) Conjunctivitis 3 (4%)
Baron—ULD-RT ^a^ [21]	2 (16%)	No toxicity	No toxicity	Dry eye 2 (16%)
Baron—MDRT ^b^ [21]	7 (29%)	3 (13%)	No toxicity	Dry eye 9 (38%) Cataract 1 (4%)

^a^ ULD-RT—ultra-low-dose radiotherapy; ^b^ MDRT—medium-dose radiotherapy; NA—not available. Note: Reported late-toxicity rates likely underestimate the true incidence, because follow-up was shorter than 5 years in most cohorts; only one study reported 5-year outcomes [12].; * Clarification: In response-adapted protocols, late toxicities occurring after dose escalation (e.g., to 20–30 Gy) are attributed to the escalated dose (Pinnix et al., grade 2 cataract post-24 Gy) [10].

## Data Availability

No new data were created or analyzed in this study.

## Supplementary Materials

The following supporting information can be downloaded at: https://www.mdpi.com/article/10.3390/cancers17172845/s1, Table S1: Quality assessment of studies using the Newcastle-Ottawa quality assessment scale; Table S2: Radiotherapy technique details; Table S3: CTCAE-harmonized acute and late toxicity by study/cohort.

## Abbreviations

The following abbreviations are used in this manuscript:

NHL	non-Hodgkin lymphoma
OAL	ocular adnexal lymphoma
MALT	mucosa-associated lymphoid tissue
3D-CRT	three-dimensional conformal radiation therapy
ULD-RT	ultra-low-dose radiotherapy
BBRT	boom-boom radiotherapy
PBT	proton beam therapy
DLBCL	diffuse large B-cell lymphoma
OS	overall survival
PFS	progression-free survival
RCTs	randomized controlled trials
CTCAE	Common Terminology Criteria for Adverse Events
MeSH	medical subject heading
PRISMA	Preferred Reporting Items for Systematic Reviews and Meta-Analyses
ORR	overall response rate
CR	complete response
LCR	local control rate
SWiM	Synthesis Without Meta-analysis
NOS	Newcastle-Ottawa scale
MZL	marginal zone lymphoma
FL	follicular lymphoma
CLL	chronic lymphocytic leukemia
MDRT	medium-dose radiotherapy
IMRT	intensity-modulated radiotherapy
CFR	conventionally fractionated radiotherapy
PS	performance status
