# Supplementary material for: Clinical Outcomes and Safety of Ultra-Low-Dose Radiotherapy for Ocular Adnexal Lymphoma: A Systematic Review
Source: Cancers (Basel). 2025 Aug 29;17(17):2845. doi: 10.3390/cancers17172845 (PMC12427289; doi:10.3390/cancers17172845)
Supplement: Supplementary file 1 [file cancers-17-02845-s001.zip › Supplementary Table S1.pdf]

Adjusted Newcastle-Ottawa Scale (NOS) Questions for ULD-RT Systematic Review

Selection (Max 4 stars)

1. Representativeness of the ULD-RT cohort

a) Truly representative: patients with indolent ocular adnexal lymphoma, treated consecutively or at a national/regional center

b) Somewhat representative: single-center, retrospective with clear criteria

c) Selected group (e.g., preselected responders, treated under compassionate use)

d) No description

2. Selection of non- ULD-RT cohort (only applies in comparative studies)

a) Drawn from the same population (e.g., MDRT arm from same institution)

b) From another institution or unmatched

c) No comparator or not reported

Note: For single-arm studies, this item is "Not applicable," and no star is awarded

3. Ascertainment of ULD-RT exposure

a) Medical/radiotherapy records or protocol-based documentation

b) Structured interview or EMR with RT details

c) Self-report

d) No description

4. Demonstration that outcome of interest (CR, relapse, toxicity) was not present at baseline

a) Baseline imaging/clinical exam before ULD-RT

b) No documentation of baseline status

Comparability (Max 2 stars)

1. Comparability of cohorts (or subgroups)

a) Controlled for histology, age, or stage (e.g., multivariable model or subgroup stratification)

b) Controlled for other potential confounders (e.g., RT technique, prior treatment, laterality)

c) No adjustment for confounding

Outcome (Max 3 stars)

1. Assessment of clinical outcome (response, toxicity, relapse)

   a) Independent ophthalmologist or radiologist, or objective criteria (CTCAE, RTOG)

   b) EMR review by treating physician

   c) Patient self-report

   d) Not described

2. Was follow-up long enough for local control or late toxicity to appear?

   a) Yes – at least 12 months median follow-up for indolent lymphoma

   b) No or unclear

3. Adequacy of follow-up

   a) Complete or all patients accounted for

   b) ≤20% lost with explanation (no bias expected)

   c) >20% lost or not described

Supplementary Table S1. Quality Assessment for Studies Using the Newcastle-Ottawa Quality Assessment Scale.

**Author (cytation)**	**Selection* (max 4)**	**Comparability** (max 2)**	**Outcome (max 3)**	**Score (max 9)**
Pinnix [10]	3	2	3	8
Yang [25]	3	1	3	7
Shelukar [26]	3	1	3	7
Pinnix [27]	3	1	3	7
Park [28]	3	1	3	7
Manta [29]	3	1	3	7
König [12]	3	2	3	8
Fasola [30]	3	1	3	7
Chelius [20]	4	1	3	8

Baron [21]	4	1	3	8

*__Selection:__ One star is missing for Pinnix [10], Yang [2], Shelukar [26], Pinnix [27], Park [28], Manta [29], König [12], and Fasola [30] due to single-arm study design. For these studies, item number 2 is marked as "Not applicable," and therefore no star is awarded.

**__Comparability:__ One star is missing for Chelius [20] and Baron [21] due to insufficient adjustment for potential confounders in the comparability domain. Yang [25], Shelukar [26], Pinnix [27], Park [28], Manta [29], and Fasola [30] are missing one star due to the absence of a control group based on radiotherapy (RT) technique.
