# Supplementary material for: Clinical Outcomes and Safety of Ultra-Low-Dose Radiotherapy for Ocular Adnexal Lymphoma: A Systematic Review
Source: Cancers (Basel). 2025 Aug 29;17(17):2845. doi: 10.3390/cancers17172845 (PMC12427289; doi:10.3390/cancers17172845)
Supplement: Supplementary file 1 [file cancers-17-02845-s001.zip › Supplementary Table S2.pdf]

Supplementary Table S2. Radiotherapy technique details.

Study (first author, year)	Modality	Technique	Beam energy	Field arrangement / Target	Immobilization	Lens shielding	Bolus	Planning (CTV→PTV; other)	Dose & fractionation	Response-adapted plan	OAR constraints	Notes
Pinnix (prospective) 2024 [10]	Photons or electrons	Conformal photons; electrons (as reported)	NR	Conjunctiva: CTV = entire conjunctiva; others: orbit (as per protocol)	NR	NR	NR	External-beam RT to 4 Gy in 2 fractions; protocol-based response assessment	4 Gy in 2 fx; response-adapted escalation permitted	Yes	NR	Prospective phase II; 43 photons / 7 electrons reported
Pinnix (retrospective) 2017 [27]	Photons or electrons	3D-CRT / electrons (institutional practice)	Electrons (MeV) reported in figure; MV photons (NR)	Conjunctiva: entire palpebral + bulbar conjunctiva; others: entire orbit	Custom thermoplastic mask	Not utilized	Used as needed for superficial disease	Plan to 4 Gy in 2 fx; target definitions as above	4 Gy in 2 fx; salvage/escalation for non-CR	Yes (salvage/escalation allowed)	NR	Text states: lens shielding not used; bolus used as necessary
Yang 2022 [25]	Photons	VMAT / conformal photons (as reported)	MV photons (NR)	CTV = orbit lesion; PTV margin 2 mm	Custom thermoplastic mask; CT-based planning	NR	NR	CTV→PTV = 2 mm; CT simulation (1 mm slices)	4 Gy in 2 fx	NR	NR	Bilateral cases treated; details of simultaneous vs sequential NR
Park (FORMAL) 2022 [28]	Electrons and photons	Electrons (conjunctiva); IMRT (retrobulbar); 3D-CRT (whole globe)	Electrons 6/9 MeV; photons 6 MV	Conjunctiva: electrons with block (1.5 cm margin); Retrobulbar: IMRT; Whole globe: 3D-CRT	Thermoplastic mask	Commercial lens shielding for conjunctival cases; lens-sparing IMRT for retrobulbar	Superflab bolus for conjunctival cases	CTV conjunctiva = whole conjunctiva; Retrobulbar PTV margin 5 mm	4 Gy in 2 fx; salvage/escalation per protocol	NR (salvage described)	Lens prioritized; IMRT used to spare lens in retrobulbar disease	Planning system: Eclipse (version reported in paper)
Manta 2024 [29]	NR	NR	NR	NR	NR	NR	NR	NR	4 Gy in 2 fx	NR	NR	Technique details not specified in text
König 2018 [12]	Photons and electrons	IMRT (50%), 3D-CRT (22%), electrons (28%)	NR	Multiple sites; orbit among extranodal sites	NR	NR	NR	NR	4 Gy in 2 fx (study-wide)	NR	NR	Technique distribution explicitly reported
Fasola 2013 [30]	Photons and electrons	Clinical setup and 3D-CRT (CT-based)	NR	Orbit vs conjunctiva field selection; emphasis on covering entire conjunctiva/orbit	Thermoplastic mask	NR	NR	CT simulation; target based on clinical + imaging; note on out-of-field relapse with limited electron field	2 Gy × 2	NR	NR	Warns against electron fields that omit parts of the conjunctiva
Shelukar 2022 [26]	NR	NR	NR	NR	NR	NR	NR	NR	4 Gy in 2 fx	NR	NR	Technique not detailed; institutional standards implied

Chelius 2021 (orbit subset) [20]	Photons / electrons / (mixed across H&N)	IMRT widely used across cohort; orbit subset technique NR	NR	Orbit subset within indolent H&N cohort	NR	Lens shielding as deemed appropriate	NR	NR	4 Gy in 2 fx (n=12) and >4 Gy for others	NR	NR	Reports lens shielding used as deemed appropriate; single bilateral cataract considered unlikely RT-related
Baron 2021 (ULD-RT) [21]	Photons / electrons / protons	Photon RT (IMRT/3D-CRT), electron RT, proton RT	NR	Orbital fields per disease site	NR	NR	NR	NR	4 Gy in 2 fx (12 courses)	NR	NR	Technique distribution across courses: photon (26; IMRT 7, 3D-CRT 19), electron (6), proton (6) — across LDRT+MDRT
Baron 2021 (MDRT) [21]	Photons / electrons / protons	Photon RT (IMRT/3D-CRT), electron RT, proton RT	NR	Orbital fields per disease site	NR	NR	NR	NR	Median 24 Gy (21–36 Gy)	NR	NR	Same center/technique mix as ULD-RT arm

Notes: NR = not reported; IMRT = intensity-modulated radiotherapy; 3D-CRT = three-dimensional conformal radiotherapy; VMAT = volumetric modulated arc therapy; CBCT = cone-beam CT; OAR = organ-at-risk;

CTV = clinical target volume; PTV = planning target volume.
