# Supplementary material for: Clinical Outcomes and Safety of Ultra-Low-Dose Radiotherapy for Ocular Adnexal Lymphoma: A Systematic Review
Source: Cancers (Basel). 2025 Aug 29;17(17):2845. doi: 10.3390/cancers17172845 (PMC12427289; doi:10.3390/cancers17172845)
Supplement: Supplementary file 1 [file cancers-17-02845-s001.zip › Supplementary Table S3.pdf]

Supplementary Table S3. CTCAE-harmonized acute and late toxicity by study/cohort.

Study / Cohort	Acute: Grade 0 (n)	Acute: Grade 1–2 (n)	Acute: Grade ≥3 (n)	Late: Grade 0 (n)	Late: Grade 1–2 (n)	Late: Grade ≥3 (n)	Source (CTCAE[a] vs CTCAE-equiv[b])	Key notes / mapping
Pinnix – prospective (column up) and retrospective (column down) study [10]		4	0		0	0	CTCAE	G1 dry eye=3; G2 dry eye=1; no ≥G3; no late events reported
		1	0		1[c]	0	CTCAE	HSV keratitis ≤G2; G2 cataract occurred after escalation to 24 Gy (attributed to escalated dose)
Yang [25]		0	0		0	0	Legacy (RTOG/EORTC)	No acute or late toxicity reported
Shelukar (patients followd by oncologist – column up; patients followed by ophtalmologist – column down) [26]		1	0		0	0	CTCAE	6% any-grade by oncologist; no ≥G2 (1/17 approximated)
			0		5	0	CTCAE	Late mild findings 5/15: dry eye=3; cataract=1; chorioretinal atrophy=1
Pinnix (Head Pinnix [27]		1	0		0	0	CTCAE	G1 dry eye=1; no late events
Park [28]		9	0		0	0	CTCAE	G1 eyelid swelling=5; G1 dry eye=1; G2 dry eye=3; events in lesions receiving salvage (escalation)
Manta [29]		0	0		3	0	CTCAE-equiv	Late mild dry eye=3/21 (14%)
König [12]		0	0		0	0	CTCAE	No acute or late toxicity reported follow-up in cohort
Fasola [30]	22	6	0		0	0	CTCAE-equiv	Acute: dry eye=1, conjunctivitis=1, edema=4; no late events
Chelius [20]		5	0		2	0	CTCAE	Acute ≤G2=5/12; Late ≤G2=2/10 (verify denominators per paper)
		66	0		48	0	CTCAE	Acute ≤G2=66/69; Late ≤G2=48/68; cataracts included among late events
Baron (ULD-RT – column up; MDRT – column down) [21]		6	0		2	0	CTCAE	Late: G1 dry eye=2 (~16%); no ≥G3
		20	0		10	0	CTCAE	Late: G1 dry eye=6, G2 dry eye=3, G1 cataract=1; no ≥G3

Notes: [a] CTCAE v5.0 where authors reported CTCAE/explicit grades.
[b] "Author-reported, CTCAE-equivalent" mapping when CTCAE was not specified (no re-grading).
[c] In response-adapted cohorts, toxicities occurring after escalation are attributed to the escalated dose (not to ULD-RT).
